# Peritonitis

**Published:** 1894-04-28

**Authors:** 


### Progress in Surgery.

### PERITONITIS.

In his Lettsomian Lectures on Peritonitis Mr. Treves[1] states that the following points may be noted with regard to the peritoneum: 1. Its surface is probably as great as that represented by the whole integument of the body. 2. It can absorb an amount equal to from 3 to 8 per cent. of the body weight. 3. It offers a limited resistance to septic organisms and their products, the resistance varying within wide limits, and being affected by age and disease. 4. No tissue in the body provides more favourable conditions for healing. 5. It does not show the same degree of vulnerability in all parts, nor are all portions of it alike in the manner in which certain lesions are responded to. The part covering the small intestine is most susceptible; the parietal layer is least so. Localised forms of peritonitis and encysted exudations are comparatively uncommon in the large area occupied by the small intestine, and are commoner in the sub-phrenic region, between the dome of the diaphragm above and the transverse colon below, to the outer side of the cæcum and in the pelvis. Extensive inflammation may occur in the hepatic and pelvic regions without inducing very alarming symptoms. Injuries of the intestine are usually fatal while lesions of the liver and bile passages are commonly slow and moderate. 6. The peritoneum appears to be possessed of great sensitiveness, peculiar and not quite comparable to that possessed by the skin; this is a matter of moment in the production of shock. The sensibility is dulled or lost when the surface is covered with lymph or inflamed. 7. A matter of great interest, though of some obscurity, concerns the condition of pressure within the abdominal area, and the effects which follow the disturbance of it. The cavity of the peritoneum is, of course, a potential space, and the creation of an actual cavity after incision will produce a disturbance of pressure relations which cannot be without effect on the sensitive tissues and their walled blood vessels within the abdominal parietes. The effect of a mere incision is often very remarkable, many cases being on record where a simple incision has been followed by complete cure in cases of tubercular peritonitis and other abdominal trouble. There are certain other points in connection with intra-peritoneal pressure which remain to be mentioned. In the first place, gravity does not appear to follow the usual lines within the abdomen. In the second place, the frequency with which the escape of fæces fails to take place in spontaneous perforation of the intestine and in punctured wounds of that viscus is somewhat remarkable. Both Treves[2] and Ziegler[3] account for this by the state of intra-abdominal pressure, and Klemm[4] asserts that the idea of the wound in the bowel being blocked up by prolapsed mucous membrane is fallacious. 8. The last point concerns the possibility of the peritoneum acquiring some degree of immunity from septic infection. This condition of immunity can be produced artificially in animals, and appears to be possible in the human subject. Other things being equal, an operation carried out within the abdomen of a person who has suffered from chronic peritonitis, or who has exhibited repeated sub-acute attacks, and whose peritoneum presents substantial adhesions, is likely to be attended with better results than when the peritoneum is found to be wholly undisturbed. In advanced cases of appendix trouble the disproportion which is often noted between the constitutional disturbance and the local condition discovered at the operation appears to be capable only of the same explanation. Mr. Treves[5] thinks the following classification of peritonitis, according to its cause, appears to be justified by our present knowledge of the subject.

1. *Peritonitis Due to Infection from the Intestine.*—Under this heading are included most cases of peritonitis associated with hernia, with intestinal obstruction, and with perforation; peritonitis due to any form of ulceration of the bowel; to enteritis; to cancerous growths of the gut, and to troubles in the appendix. There will also be included peritonitis due to inflammatory changes in the biliary canals, and some forms of peritonitis following upon operation. The micro-organism which is usually found associated with these different forms of peritonitis is the bacterium coli commune.

2. *Peritonitis Due to Infection from Without.*—This division will include puerperal peritonitis, peritonitis consequent upon inflammatory troubles in the genital organs or in the parietes of the abdomen, and some forms of peritonitis following upon operation. The micro-organisms usually associated with the varieties considered under this heading are pyogenic cocci, and notably the streptococcus pyogenes.

3. *Peritonitis Due to the Pneumococcus.*

4. *Tuberculous Peritonitis.*

5. *Peritonitis of a Doubtful Nature.*—Under this purposely indefinite heading are included—(*a*) peritonitis due to irritants; (*b*) forms of the trouble reputed to depend upon rheumatism, gonorrhœa, syphilis, Bright's disease, and alcoholism; and (c) peritonitis met with in the newly-born.

Could one single symptom be selected as the one more usually present in all cases of peritonitis, it would, says Treves,[6] probably be vomiting, but this is usually slight. It is very little in the more insidious septic cases in the aged, and in the subjects of advanced visceral disease, but it is usually marked and copious in the peritonitis following strangulated hernia; it is conspicuous in perforative peritonitis, but it is usually absent in cases of perforation of the stomach. Constipation is a conspicuous feature in peritonitis. In one hundred cases from the London Hospital the bowels are classed as "loose" in twenty-eight. From a study of these cases it would appear that, other things being equal, a better prognosis attends the cases in which the bowels are acting, and that a specially evil prognosis attends the cases marked by absolute constipation. As to lung complications, among the hundred cases there were no less than seventeen in which pleurisy or pneumonia appeared after the peritonitis set in; seven of these were examples of peritonitis of intestinal origin, five of them being cases of hernia or of perforation, and ten were cases of peritonitis starting from the pelvic organs or from sources outside the abdomen, four of the latter following a wound in the abdominal wall.

Treves[7] gives the following suggestions for treatment: 1. Absolute rest in the recumbent position appears to be the first obvious indication. 2. The old rule of giving as little food as possible by the mouth cannot be improved upon, but extremes—such as the unreasoning prohibition of food and the intemperate use of ice and iced fluids—must be avoided. The actual feeding should be carried out by rectal injections. 3. Give as little opium as is possible. The indication for it is actual pain, and not mere restlessness and misery. It should never become a feature in the routine treatment of peritonitis. In those who are dying from general peritoneal sepsis a greater sense of relief usually follows the hypodermic injection of strychnine. 4. Aperients can never be adopted in the routine treatment of peritonitis. In the larger proportion of cases this measure is entirely useless, and in septic forms it is more or less impracticable. But there is no doubt that there is within the intestine an amount of noxious matter which remains harmless so long as the viscus retains its normal condition, but which may lead to septic symptoms if certain changes are induced in the wall of the bowel, or possibly of its contents. These injurious matters can, within certain possibly narrow limits, be got rid of by the action of aperients. Of the effect of a thorough evacuation of the alimentary canal in some instances surgical experience can testify. The most successful treatment of acute obstruction of a certain grade is that which provides for a thorough evacuation of the loaded gut, and Treves is convinced of the value of the aperient treatment in the earliest stages of perityphlitis, and of the pursuit of the same measure throughout in selected instances. A further illustration of the subject from the same standpoint is afforded by that alarming intestinal condition known as "pseudo-ileus.' In the peritonitis following hernia, or associated with acute intestinal obstruction, the complete evacuation of the bowels is desirable for reasons apart from the peritonitis. The emptying of the intestines before any abdominal section is performed may be regarded as essential. On the other hand, aperients are of no avail in established general peritonitis, in septic peritonitis, and in true perforative peritonitis. 5. Blood letting may with advantage be more extensively employed, especially in the robust forms of localised peritonitis. 6. The operative treatment of suppurative peritonitis, especially when the effusion is localised, has been remarkably successful, but it is doubtful whether a single human life has been saved by surgical interference in a genuine case of peritoneal toxæmia. In generalised peritonitis, if the exudation be serous, evacuate the fluid, and close the abdomen without drainage. When the exudation is sero-purulent or purulent, irrigate with a sterile 0·6 per cent. salt solution made warm, dry the dependent parts with sponges, freely dust the involved peritoneum with iodoform powder, and introduce a long rubber fenestrated drainage-tube. In cases of plastic peritonitis free the adhesions only to such an extent as to allow the escape of imprisoned pus, dust the serous membrane with iodoform, and drain, if necessary, by strips of iodoform gauze. To close the wound sew up the peritoneum with a continuous suture of fine silk, and the rest of the parietal wound should be closed with a single row of silkworm gut sutures, which embrace all the soft parts except the serous membrane.

Barbacci[8] from a study of fourteen cases of peritonitis from perforation and by experimentation on animals has formed the following conclusions as to the etiology of peritonitis from perforation: 1. Cultures made from the peritoneal exudate in man furnish usually only a single variety of organism—the bacterium coli commune. Occasionally, however, diplococcus lanceolatus capsulatus (Fraenkel) can be shown to exist in the exudate by inoculation of mice and rabbits. 2. After extravasation of fæcal matter, the various bacteria contained therein develop equally with bacterium coli during the early stages of inflammation which is induced. But subsequently they die, whereas bacterium coli continues to thrive, and thus is susceptible of further cultivation. 3. In a number of cases one finds bacterium coli in the heart blood of persons who have died of perforative peritonitis; in others it is not to be found there, and this, coupled with the fact that it is absent from the blood of the dog when the necropsy is performed shortly after death, renders it probable that, in the first-mentioned cases, the entrance into the blood stream took place *post-mortem.* 4. Although bacterium coli is the only organism which can be cultivated out of the exudate, it cannot be regarded as the actual cause of the peritonitis. 5. The intestinal gases which pass into the peritoneal cavity with the fæces play a not inconsiderable part in originating and perpetuating the peritoneal inflammation. 6. The latter is due to two co-operating causes, each of which is by itself inoperative—namely, the extravasation of fæces and gas, and the development of germs contained in the former. The inflammation is perpetuated by the continuous irritation of the extravasated matter.

7. Death in cases of peritonitis from perforation is due to an intense intoxication of the organism, which results from the absorption of the fluid and gaseous contents of the bowel, and of toxic bacterial secretions.

Gilbert Barling[9] records ten cases of peritonitis treated by incision and drainage. Four cases died. Of these, one was a case of appendicitis, one of localised suppurative peritonitis presenting in the epigastrium, with hepatic abscess, and two were cases of tuberculous peritonitis.

Dr. Syms[10] states that according to Koenig's investigations it appears that about 60 per cent. of patients suffering from tuberculous peritonitis whose abdomens had been opened and closed without further interference were apparently cured, and that in 25 per cent. the cure was permanent, assuming that two years' immunity meant permanent cure. M. Demailer[11] quotes a case of non-exudative tubercular peritonitis treated by incision and drainage. The case did well for a month, then bad symptoms reappeared concomitantly with a fæcal fistula, and the patient died two months after the operation. He remarks that the non-exudative peritonitis, contrary to the exudative forms, should be looked upon as non-operable.

[1] Lancet, Feb. 3, 1894, and British Med. Journ., Feb. 3, 1894. [2] "An Inquiry into the Repairing of Injuries of the Intestines," London, 1812, pp. 16, 18 et seq. [3] "Uber die Intestinale Form der Peritonitis," Munich, 1893. [4] Deut. Zeit. für Chir., 1892. [5] Brit. Med. Journ., Feb. 17, 1894, p. 341. [6] Lancet, March 3, 1894. [7] Ibidem. [8] Brit. Med. Journ., Dec. 23, 1893. [9] Brit. Med. Journ., Jan. 20, 1894, p. 122. [10] Annals of Surgery, Jan., 1894. [11] Med. Record, Feb. 3, 1894, p. 149.

